# Tumour growth rates of prostate cancer during active surveillance: is there a difference between MRI-visible low and intermediate-risk disease?

**DOI:** 10.1259/bjr.20210321

**Published:** 2021-07-08

**Authors:** Francesco Giganti, Clare Allen, Vasilis Stavrinides, Armando Stabile, Aiman Haider, Alex Freeman, Nora Pashayan, Shonit Punwani, Mark Emberton, Caroline M Moore, Alex Kirkham

**Affiliations:** 1 Department of Radiology, University College London Hospital NHS Foundation Trust, London, UK; 2 Division of Surgery & Interventional Science, University College London, London, UK; 3 Department of Urology, University College London Hospital NHS Foundation Trust, London, UK; 4 Department of Urology and Division of Experimental Oncology, Vita-Salute San Raffaele University, Milan, Italy; 5 Department of Pathology, University College London Hospital NHS Foundation Trust, London, UK; 6 Department of Applied Health Research, University College London, London, UK; 7 Centre for Medical Imaging, University College London, London, UK

## Abstract

**Objectives:**

The aim of this study was to evaluate the changes in lesion volume on serial multiparametric magnetic resonance (mpMRI) during active surveillance for prostate cancer.

**Methods:**

A total of 160 patients with a targeted biopsy-confirmed visible lesion on mpMRI, stratified by low- and intermediate-risk disease (Gleason Grade Group 1 vs Gleason Grade Group 2), were analysed. The % change per year was calculated using the formula: *[(final volume/initial volume) exp (1/interval between scans in years)]-1*.

**Results:**

There was no significant difference in the annual median percentage change between Gleason Grade Group 1 (18%) and Gleason Grade Group 2 (23%) disease (p = 0.16), and between ≤ 10% (23%) and > 10% (22%) of Gleason pattern 4 (p = 0.78).

Assuming a spherical lesion, these changes corresponded to annual increases in mean tumour diameter of 6% and 7% for Gleason Grade Group 1 and Gleason Grade Group 2 respectively, which may be less than the interscan variability of serial mpMRI.

**Conclusion:**

In an active surveillance cohort, we did not see a significant difference in the annual growth rate of Gleason Grade Group 1 and 2 tumours.

**Advances in knowledge:**

In patients on active surveillance, the measured growth rates for visible tumours in Gleason Grade Groups 1 and 2 were similar. The annual growth rate was small in most cases and this may have implications for the MRI follow-up interval in active surveillance.

## Introduction

There has been growing interest in the use of multiparametric magnetic resonance (mpMRI) for active surveillance (AS) in low- to intermediate-risk prostate cancer over the last decade, both to exclude undetected high-grade tumour at entry, and to monitor size and conspicuity.^1^

We know that a suspicious lesion on mpMRI is seen in two-thirds of males otherwise suitable for AS^1^ and that by performing mpMRI before biopsy we can target MR-visible lesions with suspicious radiological features (or showing signs of radiological progression during AS), detecting a higher percentage of patients with clinically significant prostate cancer and lowering the diagnosis of clinically insignificant disease.^2^

Current guidelines for reporting MRI in this context distinguish between ‘stable’ or ‘increased’ tumour volume or conspicuity^3^ but they need to be refined: we cannot expect the majority of tumours to stay unchanged in volume over many years. Estimates of normal growth rate are needed so that we can identify outliers potentially requiring treatment. However, almost all our estimates of prostate cancer growth come from studies of prostate-specific antigen (PSA) kinetics, rather than imaging.^4^

In this study, we evaluated the changes in lesion volume for MR-visible lesions in our AS cohort over time and stratified them according to Gleason Grade Group.

## Methods and materials

Our MRI-guided AS cohort at University College London Hospital (UCLH) includes patients who have had a prostate mpMRI and a biopsy-confirmed low- to intermediate-risk prostate cancer (*i.e.* Gleason Grade Group ≤ 2 and PSA ≤ 20 ng ml^−1^) as per UK national guidelines.^5^

At UCLH, all clinical records and MR images are routinely reviewed as part of an audit performed for the internal evaluation of the AS service and no institutional review board approval was required.

The cohort presented in this study is a consecutive series of patients meeting the following inclusion criteria: i) a targeted biopsy at entry into or during AS (if multiple targeted biopsies, the first one was used in the analysis); ii) at least two MR scans (the first one being the closest to the targeted biopsy) with a visible lesion, and in case of more than two scans we analysed the first and last scans.

### Image protocol and analysis

In this study, two 1.5T (Symphony or Avanto, Siemens) and one 3T (Achieva, Philips) MR scanners were used. The protocol comprised *T*_2_ weighted imaging (*T*_2_WI), diffusion-weighted imaging (DWI) (including multiple *b* values of 0, 150, 500, 1,000 s/mm^2^ for the apparent diffusion coefficient - ADC - map and dedicated high *b* value sequences: 1,400 s/mm^2^ for 1.5T or 2,000 s/mm^2^ for 3T) and dynamic contrast-enhanced (DCE) sequences, with no endorectal coil. All acquisitions, including the ADC map and DCE sequences, were used in this study.

The index lesion volume was measured by planimetry on the sequence best showing the tumour (as reported in Table 1) on baseline and follow-up scans (but not necessarily on the same MR scanner) by a dedicated genitourinary radiologist (FG, reporting > 2,000 prostate MR scans per year) who was blinded to all clinical and pathological data, using dedicated software (MIM^®^ Symphony Dx, Cleveland, OH). All lesions were scored according to the Prostate Imaging Data and Reporting System (PI-RADS) v. 2.1 recommendations.

**Table 1.** Clinical and MR characteristics at baseline

	Overall	Gleason Grade Group 1	Gleason Grade Group 2
	(*n* = 160)	(*n* = 84)	(*n* = 76)
Median age (years)	63 [59-69]	65 [59-69]	63 [58-69]
				PSA density (ng/ml/ml)	0.15 [0.1–0.2]	0.15 [0.1–0.19]	0.14 [0.09–0.21]
PI-RADS lesions			
3	44 (28%)	28 (33%)	16 (21%)
4	106 (66%)	51 (61%)	55 (72%)
5	10 (6%)	5 (6%)	5 (7%)
Lesion location			
Peripheral zone	134 (84%)	68 (81%)	64 (84%)
Transitional zone	26 (16%)	16 (19%)	12 (16%)
Best visible sequence			
*T*_2_WI	48 (30%)	28 (33%)	20 (26%)
DWI ^a^	57 (36%)	27 (32%)	27 (36%)
DCE	55 (34%)	29 (35%)	29 (38%)

DCE: dynamic contrast enhanced; DWI: diffusion-weighted imaging; PI-RADS: Prostate Imaging Data and Reporting System;PSA: Prostate Specific Antigen; *T*_2_WI: *T*_2_ weighted imaging.

Data are medians with interquartile ranges in brackets. Percentage in parentheses.

a For lesions best visible on DWI, these were always delineated on the high *b* value sequence and not on the ADC map.

### Statistical analysis

Data are presented as median and interquartile ranges (IQR).

The % change per year was calculated using the standard following formula for deriving the compound annual growth rate^6^ :

[(final volume/initial volume) exp (1/interval between scans in years)]-1

The Mann–Whitney test was used to assess differences between groups.

## Results

From our initial cohort of 553 patients undergoing AS, 266 (48%) did not have a targeted biopsy, 106 (19%) had no visible lesions on mpMRI, 15 (3%) had a lesion visible only on oneMR scan and 6 (1%) had Gleason Grade Group ≥ 3 at targeted biopsy.

For those patients who did not have a targeted biopsy, the reason was that biopsies were recommended at the discretion of the treating physician. The recommendation was based either on the suspicion of progression on MRI, or on adverse PSA kinetics without MRI changes. Some patients, particularly those with Gleason Grade Group 2 disease at the outset of surveillance who could have chosen active treatment, did not wish to have a further biopsy before proceeding to treatment.

Therefore, a total of 160 patients were finally included (study period: January 2007 – November 2019), 84 (52%) of which had Gleason Grade Group 1 and 76 (48%) had Gleason Grade Group 2 disease at targeted biopsy. 130 (81%) patients had at least an additional biopsy.

Baseline clinical and MRI data are shown in Table 1.

Median interval between first and last scan was 38 months (IQR: 24–57 months).

Median follow-up for Gleason Grade Group 1 and Gleason Grade Group 2 was 41 (IQR: 24–61) months and 33.5 (IQR: 22–57) months, respectively (*p* = 0.59).

**Figure 1.**
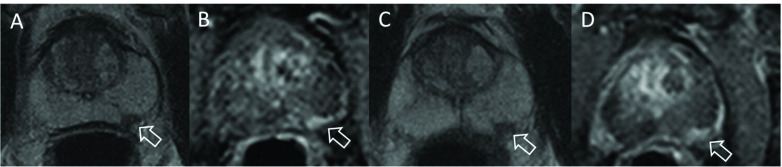
Axial *T*_2_ weighted (**A, C**) and dynamic-contrast enhanced (**B, D**) acquisitions of a patient with prostate cancer on active surveillance. The patient had Gleason Grade Group 2 disease at targeted biopsy of the lesion in the left peripheral zone (arrow) at baseline (**A, B**), with a lesion volume by planimetry on *T*_2_ weighted imaging (**A**) of 0.12 cc. After 3 years on active surveillance, tumour volume on *T*_2_ weighted imaging (**C**) was 0.39 cc, with an estimated annual growth rate of 45%.

**Figure 2.**
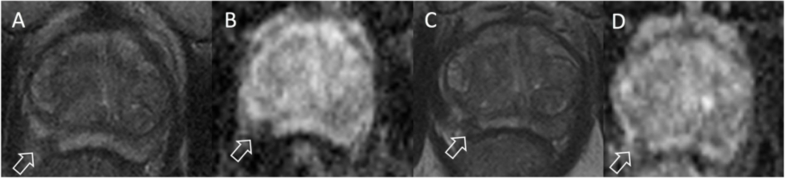
Axial *T*_2_ weighted (**A, C**) and apparent diffusion coefficient maps from diffusion-weighted imaging (**B, D**) acquisitions of a patient with prostate cancer on active surveillance. The patient had Gleason Grade Group 1 disease at targeted biopsy of the lesion in the right peripheral zone (arrow) at baseline (**A, B**), with a lesion volume by planimetry on the ADC map (**B**) of 0.34 cc. After 3 years of active surveillance, tumour volume on the ADC map (**D**) was 0.56 cc, with an estimated annual growth rate of 62%.

There was no significant difference in the percentage change per year between Gleason Grade Group 1 (median: 18%; IQR: 2–37) and Gleason Grade Group 2 (median: 23%; IQR: 7–38) disease (*p* = 0.16), as shown in Table 2 and Figures 1 and 2.

**Table 2.** Change per year (%) stratified by MRI-visible Gleason Grade Group (Group 1 vs Group 2) and Gleason pattern 4 at targeted biopsy (≤ or > 10%).

	Gleason Grade Group 1 (*n* = 84)	Gleason Grade Group 2 (*n* = 76)		p
Change per year (%)	18 [2 - 37]	23 [7 - 38]		0.16
	
	-	≤10% pattern 4 * (*n* = 32)	>10% pattern 4 * (*n* = 25)	p
Change per year (%)	-	23 [6 - 39]	22 [6 - 34]	0.78

Data are medians with interquartile ranges in brackets.

a data available for 57 patients

For Gleason Grade Group 2, when we applied a cut-off of 10% of Gleason pattern 4 (data available for 57 patients), we observed no significant difference between patients with Gleason pattern 4 ≤ 10% (*n* = 32; median change: 23%; IQR: 6–39) and those with Gleason pattern 4 > 10% (*n* = 25; median change: 22%; IQR: 6–34) (*p* = 0.78). (Table 2)

## Discussion

There are two main imaging parameters that should be taken into account during AS of small tumours.

The first is *conspicuity*: previously invisible or equivocal foci may become more visible, triggering treatment.^7^

The second key parameter is *tumour volume*, and in our study we found that there was not a marked difference in the annual change between Gleason Grade Group 1 and Gleason Grade Group 2 MR-visible tumours, although we should keep in mind that mpMRI can overestimate volume for low- and intermediate-risk disease, as small lesions are often surrounded by areas of inflammation/atrophy that can mimic low-grade tumour.^8^

Conversely, there is also evidence that MRI can underestimate the volume of prostate cancer, especially in low grade disease.^9,10^ In particular, Sun and colleagues^10^ analysed the comparative effectiveness of different MRI sequences for the estimation of the index lesion volume compared with volume measured on whole-mount pathology. The lesion volume was underestimated on *T*_2_WI (55%), ADC maps (59%), and DCE images (18%) compared with histopathology.

However, the important parameter in AS is *change,* and we still do not know which sequence has the lowest variability on multiple follow-up scans.

This study has two main limitations: the first is that the order of the scans was known by the Radiologist, potentially biasing the measurement of volume, and the second is that the wide interquartile ranges may in part reflect interscan variability. Other limitations include the different MR scanners and magnet strengths used, the variable follow-up and the lack of comparison between change in size and pathological upgrading. Another limitation is that we did not undertake any analysis of the initial size or change in size with respect to upgrading, but this represents fertile ground for future research.

The most important clinical implication of this study may be in determining the MRI follow-up interval in AS. If we assume a spherical lesion, the annual increases of 18% and 23% in Grade Group 1 and Grade Group 2 tumours correspond to annual increases in mean tumour diameter of 6% and 7% respectively, which is considerably less than the interscan variability of serial mpMRI^11^: annual scans may well be too frequent to detect tumour change in most patients.

## Conclusion

In a study with median follow-up of 38 months, we noted annual changes in volume of 18% for MR-visible Gleason Grade Group 1 and 23% for MR-visible Gleason Grade Group 2 disease, though with each grade the range was wide.

## References

[1] Schoots IG, Petrides N, Giganti F, Bokhorst LP, Rannikko A, Klotz L, et al . Magnetic resonance imaging in active surveillance of prostate cancer: a systematic review. Eur Urol 2015; 67: 627–36. doi: 10.1016/j.eururo.2014.10.050 25511988

[2] Kasivisvanathan V, Rannikko AS, Borghi M, Panebianco V, Mynderse LA, Vaarala MH, et al . MRI-Targeted or standard biopsy for prostate-cancer diagnosis. N Engl J Med 2018; 378: 1767–77. doi: 10.1056/NEJMoa1801993 29552975 PMC9084630

[3] Moore CM, Giganti F, Albertsen P, Allen C, Bangma C, Briganti A, et al . Reporting magnetic resonance imaging in men on active surveillance for prostate cancer: the precise Recommendations—A report of a European school of oncology Task force. Eur Urol 2017; 71: 648–55. doi: 10.1016/j.eururo.2016.06.011 27349615

[4] van den Bergh RCN, Roemeling S, Roobol MJ, Wolters T, Schröder FH, Bangma CH . Prostate-Specific antigen kinetics in clinical decision-making during active surveillance for early prostate Cancer—A review. Eur Urol 2008; 54: 505–16. doi: 10.1016/j.eururo.2008.06.040 18585845

[5] National Institute for Health and Care Excellence (NICE). Prostate cancer diagnosis and management (NICE Guideline 131). 2019. Available from: https://www.nice.org.uk/guidance/ng131 [Accessed 1st February 2021]. 31393679

[6] https://en.wikipedia.org/wiki/Compound_annual_growth_rate [Accessed 20th April 2021].

[7] Morgan VA, Parker C, MacDonald A, Thomas K, deSouza NM, et al . Monitoring tumor volume in patients with prostate cancer undergoing active surveillance: is MRI apparent diffusion coefficient indicative of tumor growth? American Journal of Roentgenology 2017; 209: 620–8. doi: 10.2214/AJR.17.17790 28609110

[8] Bratan F, Niaf E, Melodelima C, Chesnais AL, Souchon R, Mège-Lechevallier F, et al . Influence of imaging and histological factors on prostate cancer detection and localisation on multiparametric MRI: a prospective study. Eur Radiol 2013; 23: 2019–29. doi: 10.1007/s00330-013-2795-0 23494494

[9] Kramer M, Spohn SKB, Kiefer S, Ceci L, Sigle A, Oerther B, et al . Isotropic expansion of the Intraprostatic gross tumor volume of primary prostate cancer patients defined in MRI—A correlation study with whole Mount histopathological information as reference. Front Oncol 2020; 10: 596756. doi: 10.3389/fonc.2020.596756 33330088 PMC7719800

[10] Sun C, Chatterjee A, Yousuf A, Antic T, Eggener S, Karczmar GS, et al . Comparison of T2-weighted imaging, DWI, and dynamic contrast-enhanced MRI for calculation of prostate cancer index lesion volume: correlation with Whole-Mount pathology. American Journal of Roentgenology 2019; 212: 351–6. doi: 10.2214/AJR.18.20147 30540213

[11] Giganti F, Moore CM, Punwani S, Allen C, Emberton M, Kirkham A . The natural history of prostate cancer on MRI: lessons from an active surveillance cohort. Prostate Cancer Prostatic Dis 2018; 21: 556–63. doi: 10.1038/s41391-018-0058-5 30038388

